# Editorial: Emerging talents in B cell biology: 2022

**DOI:** 10.3389/fimmu.2023.1335263

**Published:** 2023-11-29

**Authors:** Michael Zemlin, Rudi W Hendriks, Harry W Schroeder

**Affiliations:** ^1^ Departmen for General Pediatrics and Neonatology, Saarland University Medical Center, Homburg, Saarland, Germany; ^2^ Department of Pulmonary Medicine, Erasmus Medical Care, University Medical Center, Rotterdam, Netherlands; ^3^ Division of Clinical Immunology and Rheumatology, Department of Medicine, University of Alabama at Birmingham Heersink School of Medicine, Birmingham, AL, United States

**Keywords:** emerging talents, B cell biology, Breg, glycosylation, exosomes, hypoxia

This Research Topic on *Emerging Talents in B cell Biology 2022* highlights the crucial role of young investigators in the field of B cell biology. These newcomers to the field often perform key research and write excellent dissertations or theses that are not distributed to a broader scientific community. Multiple factors might prevent the publication of student research. For example, the process of participating in a peer review process might appear daunting, and moving to a new position might hamper the publication of a project that was accomplished in another group. However, many outstanding pioneers in the field of B cell biology, including Nobel prize laureates Suzumi Tonegawa and Georges Kohler, have published pivotal manuscripts quite early in their careers.

This Research Topic presents the work of outstanding young researchers in the field of B cell biology who began their publication activities within the last three years. These newcomers represent four different countries. Due to self-perceived equal contributions by more than one first author, eight first authors are reported among the four manuscripts. It is noteworthy that the first authors in four are women. Moreover, the majority of co-authors are also female, and four of the seven senior authors are male. This cohort facilitates current awareness of increasing female participation in academia and in the biomedical field in particular: although further interpretation is beyond our area of competence.

The Research Topic includes one original article and three review articles and spans a large area of current hot topics in B cell biology:

Zheremyan et al. present original research aiming to explore stimuli that have the potential to drive the differentiation of B cells with a regulatory phenotype *in vitro*. The authors argue that research on the Breg function is limited by the low number of blood samples and higher numbers of Breg that are required for therapeutic adoptive cell transfer for diseases associated with excessive immune activation. Thus, they used various cocktails to stimulate enriched human CD19+ B cells and tested the Breg ratio, viability, proliferation rates, and the expression of major molecules involved in Breg-mediated immunoregulation such as IL-10 and IL-35, as well as *in vitro* suppressive activity. This helps us better understand the differentiation of Bregs. Moreover, this work offers interesting experimental models and might ultimately enable the development of novel strategies for using Bregs in therapies against allergies, graft-versus-host disease, and autoimmune diseases.

A touch of enthusiasm accompanies the title “*Revolutionizing anti-tumor therapy: unleashing the potential of B cell-derived exosomes*” by Xiong et al. The authors provide a mini-review on the role of B-cell derived exosomes in the tumor microenvironment. Since the discovery of B cell-derived exosomes in 1996 (1), it has become clear that a complex symphony of signals can induce exosome production and composition in B cells. Exosome cargo can modify immune responses to tumors, including the delivery of therapeutic agents. Moreover, exosomes can induce antigen responses by expressing antigens on their surface. The therapeutic use of exosomes presents a range of challenges but has the potential to revolutionize targeted anti-tumor therapy, as suggested by Xiong et al. As a result, this technology is currently under intensive investigation by many research groups. This review article may help both the B cell and the onco-immunology communities to gain a quick and thorough overview of this rapidly developing field of research.

Trzos et al. point to another fascinating mechanism in B cell biology: the post-translational modification of key molecules. N-glycosylation of immune cell receptors and IgG molecules is a key regulator of the immune system. Given that altered profiles of N-glycosylation contribute to disease progression and remission in autoimmune disorders, this mechanism deserves attention. In this review article, the authors summarize current knowledge of the regulation of B cell development, pre-B cell receptor (pre-BCR) folding, BCR-mediated signaling, and – one of the most fascinating observations – the influence of N-glycosylation on the function of IgG. Although N-glycosylation of immunoglobulins has been known for decades, experts in glycoimmunology are still struggling to establish reliable therapeutic uses for this key mechanism. By helping visualize the current concepts of glycoimmunology through the use of highly instructive figures, Trzos et al. clarify a confusing field, and articles like this help translate basic research into clinical application.

Koers et al. provide evidence that oxygen pressure in germinal centers (GC) is a critical regulator of human B cell differentiation and Ig class switch recombination towards IgG. Memory B cells and antibody producing cells are generated during the GC response, which involves complex mechanisms that regulate the cycling of B cells through the light and dark zones, proliferation, affinity maturation, clonal selection, and cellular differentiation. Given that variations in pO2 profoundly affect the cellular metabolism of lymphocytes, the authors aimed to study the effects of pO2 on B cell differentiation. To this end, they use an *in vitro* system in which the interactions of B cells with follicular T helper cells are mimicked by B cell cultures on CD40L-expressing 3T3 fibroblasts in the presence of IL-4 and IL-21, under different pO2 conditions. By an in-depth analysis of B cell phenotype, signaling, and metabolism, Koers et al. show that under hypoxia, a unique population of CD27-high B cells is generated, which has an enhanced capacity to differentiate into class-switched antibody secreting cells. This would support the notion that during *in vivo* B cell cycling between the GC light zone and GC dark zone, the dynamic variation in pO2 forms another intriguing regulatory layer for human B cell differentiation.

This small but fine Research Topic highlights the great quality of research that young researchers perform in the field of B cell biology. We wish the authors of articles included in this Research Topic of *Emerging Talents in B cell Biology 2022* success in their careers.

## Author contributions

MZ: Writing – original draft, Writing – review & editing. RH: Writing – review & editing. HS: Writing – review & editing.
